# Massive Preperitoneal Hematoma after a Subcutaneous Injection

**DOI:** 10.1155/2016/7013708

**Published:** 2016-09-21

**Authors:** Hideki Katagiri, Kentaro Yoshikawa, Alan Kawarai Lefor, Tadao Kubota, Ken Mizokami

**Affiliations:** ^1^Department of Surgery, Tokyo Bay Urayasu Ichikawa Medical Center, 3-4-32 Todaijima, Urayasu, Chiba 279-0001, Japan; ^2^Department of Surgery, Jichi Medical University, 1-3311 Yakushiji, Shimotsuke, Tochigi 329-0498, Japan

## Abstract

Preperitoneal hematomas are rare and can develop after surgery or trauma. A 74-year-old woman, receiving systemic anticoagulation, developed a massive preperitoneal hematoma after a subcutaneous injection of teriparatide using a 32-gauge, 4 mm needle. In this patient, there were two factors, the subcutaneous injection of teriparatide and systemic anticoagulation, associated with development of the hematoma. These two factors are especially significant, because they are widely used clinically. Although extremely rare, physicians must consider this potentially life-threatening complication after subcutaneous injections, especially in patients receiving anticoagulation.

## 1. Introduction

Preperitoneal hematomas have been rarely reported to date and can develop after surgical procedures or trauma. In patients receiving systemic anticoagulation, they can also develop spontaneously. Here, we present a very rare case of a patient who developed a preperitoneal hematoma after a subcutaneous injection of teriparatide.

## 2. Case Report

A 74-year-old woman, admitted for planned total knee arthroplasty, was seen in consultation by the general surgery service because of a massive preperitoneal hematoma. The patient had a past medical history of Graves' disease treated surgically at the age of 20, mitral valvuloplasty for mitral regurgitation 17 years previously, and pacemaker implantation for sick sinus syndrome 16 years previously. The patient was treated with warfarin after valvuloplasty because of a previous left atrial thrombus.

**Figure 1:**
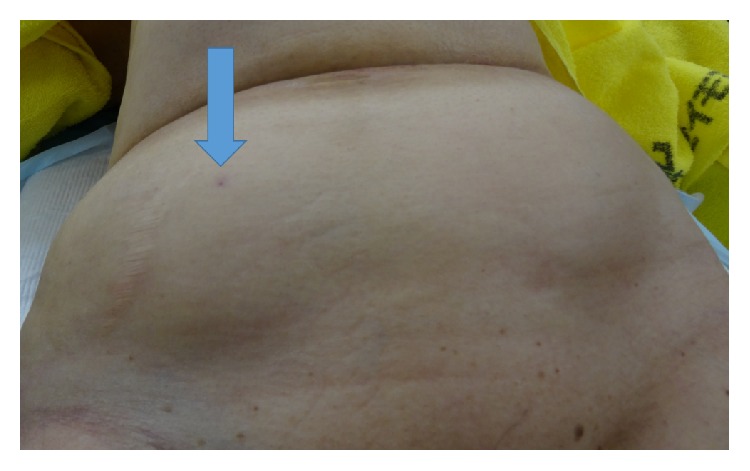
The right lower abdomen, showing a distended right lower quadrant with a central injection site (arrow).

**Figure 2:**
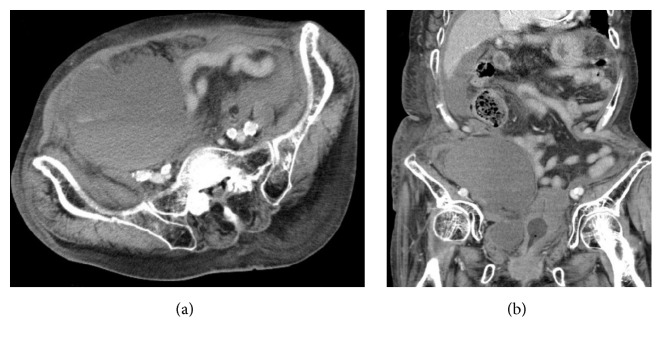
Abdominal computed tomography scans with intravenous contrast showing axial and coronal views. A massive preperitoneal hematoma is present beneath the right hypogastric vessels with hemoperitoneum.

Five days prior to consultation, she was admitted to the orthopedic surgery service for a planned total knee arthroplasty. Since she was currently receiving warfarin, the warfarin was stopped and heparin given as bridging anticoagulation therapy. Anticoagulation was well controlled in the outpatient setting, with a prothrombin time international normalized ratio (PT-INR) of 2.00 before admission. Three days prior to consultation, she began receiving subcutaneous teriparatide using a 32-gauge, 4 mm needle. After the first injection of teriparatide in the right lower abdomen, she noticed right sided back pain. On that day, the PT-INR was 2.00; however, the activated partial thromboplastin time (aPTT) was prolonged at >100 seconds. On the day of consultation, she became hypotensive which responded to an intravenous bolus of normal saline. She denied any history of abdominal trauma prior to admission. On physical examination, her right lower quadrant was distended and tender, with an injection scar in the central area (Figure 1). The aPTT was continuously prolonged at >100 seconds. Abdominal computed tomography (CT) scan with intravenous contrast was obtained, which revealed a massive preperitoneal hematoma and hemoperitoneum (Figures 2(a) and 2(b)). No apparent extravasation was detected; however, bleeding from the hypogastric vessels was suspected based on the location of the hematoma. Based on these findings, the general surgery service was consulted. A massive preperitoneal hematoma with hemoperitoneum due to subcutaneous teriparatide injection was suspected. Since the patient was hypotensive, urgent surgery was undertaken, and McBurney's incision was made. When the preperitoneal space was opened, uncoagulated blood spontaneously flowed out. Blood in the peritoneal cavity had not coagulated and was easily aspirated. There was no apparent bleeding site in the abdominal cavity. We ligated the right hypogastric vessels and closed the wound. Her postoperative course was uneventful and she underwent total knee arthroplasty 10 days later.

## 3. Discussion

Preperitoneal hematoma has rarely been reported previously. In the present patient, there were two factors, subcutaneous injection of teriparatide and systemic anticoagulation, associated with this condition. These two factors are especially significant, because they are widely used clinically.

Subcutaneous injections, for medications such as insulin, are widely used. Since diabetes mellitus is a common disease, the number of people receiving subcutaneous insulin is increasing [1]. Even though this is commonly used, complications after subcutaneous injections are thought to be rare. Erythema, pruritus, and lipohypertrophy are relatively common problems associated with insulin injections [1]. Although subcutaneous hematomas, or abdominal wall hematomas after insulin injection, have been reported [2, 3], a massive preperitoneal hematoma after a subcutaneous injection has not been reported. To the best of our knowledge, this is the first report of a preperitoneal hematoma after a subcutaneous injection.

In this patient, the subcutaneous injection of teriparatide, a subcutaneously administered agent for the treatment of osteoporosis [4], was given with a very thin, short needle (32 G, 4 mm). This is the same sized needle used for subcutaneous insulin injections. This means that subcutaneous injection of insulin can also potentially cause this serious complication. While the use of heparin was surely a part of the genesis of this complication in this patient, the fact that a small gauge needle can cause this life-threatening complication is notable. Furthermore, Pace et al. reported that low molecular weight heparin can cause a fatal spontaneous extraperitoneal hematoma [5]. Physicians have to consider hemorrhagic complications in patients receiving heparin. Although the actual site of bleeding was not seen intraoperatively, we believe that the hypogastric vessels were the origin, based on the history of developing a backache just after the subcutaneous injection, the imaging findings, and location of the hematoma. Within three days, the hematoma had spread not only in the preperitoneal space but also into the peritoneal cavity. This was due to the administration of heparin and resulting anticoagulation. In general, subcutaneous heparin administration does not need monitoring; however, as highlighted by events in this patient, intravenous administration of unfractionated heparin requires close monitoring of aPTT. Although the necessity of bridging anticoagulation is not clearly defined [6], close monitoring and adjustment of aPTT are required when necessary.

Although extremely rare, physicians must consider this potentially life-threatening complication after subcutaneous injections, especially in patients undergoing anticoagulation.
